# Influence of Inhibition of COX-2-Dependent Lipid Metabolism on Regulation of UVB-Induced Keratinocytes Apoptosis by Cannabinoids

**DOI:** 10.3390/biom12060842

**Published:** 2022-06-17

**Authors:** Piotr Wójcik, Michał Biernacki, Natalia Domian, Neven Žarković, Elżbieta Skrzydlewska

**Affiliations:** 1 Department of Analytical Chemistry, Medical University of Bialystok, 15-089 Białystok, Poland; michal.biernacki@umb.edu.pl (M.B.); elzbieta.skrzydlewska@umb.edu.pl (E.S.); 2 Department of Histology and Cytophysiology, Medical University of Bialystok, 15-089 Białystok, Poland; natalia.domian@umb.edu.pl; 3 Laboratory for Oxidative Stress (LabOS), Rudjer Boskovic Institute, Bijenicka 54, HR-1000 Zagreb, Croatia; neven.zarkovic@irb.hr

**Keywords:** apoptosis, cyclooxygenase, keratinocytes, cannabinoids, anandamide, cannabidiol, prostaglandin derivatives

## Abstract

Inflammation and apoptosis are regulated by similar factors, including ultraviolet B (UVB) radiation and cannabinoids, which are metabolized by cyclooxygenase-2 (COX-2) into pro-apoptotic prostaglandin derivatives. Thus, the aim of this study was to evaluate the impact of cyclooxygenase-2 inhibition by celecoxib on the apoptosis of keratinocytes modulated by UVB, anandamide (AEA) and cannabidiol (CBD). For this purpose, keratinocytes were non-treated/treated with celecoxib and/or with UVB and CBD and AEA. Apoptosis was evaluated using microscopy, gene expressions using quantitate reverse-transcriptase polymerase chain reaction; prostaglandins using liquid chromatography tandem mass spectrometry and cyclooxygenase activity using spectrophotometry. UVB enhances the percentage of apoptotic keratinocytes, which can be caused by the increased prostaglandin generation by cyclooxygenase-2, or/and induced cannabinoid receptor 1/2 (CB1/2) expression. AEA used alone intensifies apoptosis by affecting caspase expression, and in UVB-irradiated keratinocytes, cyclooxygenase-2 activity is increased, while CBD acts as a cytoprotective when used with or without UVB. After COX-2 inhibition, UVB-induced changes are partially ameliorated, when anandamide becomes an anti-apoptotic agent. It can be caused by observed reduced generation of anandamide pro-apoptotic derivative prostaglandin-ethanolamide by COX. Therefore, products of cyclooxygenase-dependent lipid metabolism seem to play an important role in the modulation of UVB-induced apoptosis by cannabinoids, which is particularly significant in case of AEA as inhibition of cyclooxygenase reduces the generation of pro-apoptotic lipid mediators and thus prevents apoptosis.

## 1. Introduction

Apoptosis is a cell death mechanism that has developed evolutionarily in every multicellular organism as a response to the fact that some cells are unnecessary or need to be removed during the organism’s development. Nevertheless, the process needs to be precisely controlled, as both insufficient and excessive apoptosis may lead to various pathological conditions. Moreover, similar mechanisms that control apoptosis, such as modulation of cytokine synthesis or the action of endocannabinoids, are also involved in the regulation of inflammation, which suggests crosstalk between inflammation and apoptosis. However, inhibition of apoptosis may lead to the accumulation of abnormal cells, or even to the development of cancer, whereas increased apoptosis leads to malfunction in different body systems and tissues [1].

Traditionally, it is believed that apoptosis can occur through one of the following three major pathways: intrinsic, associated with an increased permeability of mitochondrial membranes resulting in the release of cytochrome C and the consequent activation of caspases, especially caspase 9; extrinsic, in which the activation of death receptors by the so-called death ligands leads to the formation of complexes that activate caspases (especially caspase 8); and endoplasmic reticulum (ER) stress-induced, which activates various proteins, in turn leading to caspase 2 activation [2]. All these pathways can occur simultaneously, influencing each other. Many factors, both endogenous and exogenous, modulate the apoptotic process. The most frequently mentioned exogenous factors are interleukins and other cytokines, both pro- and anti-apoptotic. The major pro-apoptotic cytokine is tumor necrosis factor α (TNFα), which is the primary activator of the extrinsic cell death pathway [3,4]. Nevertheless, TNFα is also one of the most important pro-inflammatory cytokines. Moreover, while regulating cellular metabolism, cytokines also affect other apoptotic pathways, e.g., the endoplasmic reticulum (ER) stress pathway is activated by interleukin 22 (IL-22) [5]. On the other hand, interferon γ (IFNγ) has the ability to activate the extrinsic pathway [6].

However, the course of the apoptotic process is also significantly influenced by lipid mediators generated as a result of reactive oxygen species (ROS)- and enzyme-dependent metabolism of phospholipids. Among them, the products of cyclooxygenase (COX1/2) activity are especially important. The main substrate of COX1/2 is arachidonic acid (AA), released from phospholipids by phospholipase A2 (PLA2) and from endocannabinoids mainly by fatty acid amide hydrolase (FAAH) and (monoacylglycerol lipase) MAGL [7], metabolized to prostaglandin G2 (PGG2), which is then reduced to prostaglandin H2 PGH2, and rapidly converted to other prostaglandins (e.g., PGE2, PGF2, PGD2, PGI2) and thromboxanes (e.g., thromboxane A2) via specific prostaglandin and thromboxane synthases [8].

The products of COX1/2 metabolism, such as D series prostaglandins, are pro-apoptotic, whereas E series prostaglandins are anti-apoptotic [9,10]. PGE2 has been shown to stimulate B-cell lymphoma 2 (Bcl2) expression, while PGD2 has the opposite effect. It is important to note that Bcl2 is the main anti-apoptotic protein. It is in complex with pro-apoptotic (bcl-2-like protein 4) Bax and blocks its action, while the released and activated Bax moves to the mitochondrial membrane, causing the formation of pores and leading to the release of pro-apoptotic factors, especially cytochrome C, endonuclease G, Smac (AIF)/Diablo inducer, and Omi/HtrA2 serine proteases [11]. AA is also metabolized by LOXs and cytochrome P450, generating, e.g., hydroxyeicosatetraenoic acid (HETE) [12]. Products of COX-2-dependent AA metabolism are also important pro-inflammatory mediators.

Important lipid mediators participating in the regulation of cellular metabolism are cannabinoids, including endocannabinoids (among which 2-acyloglycerol (2-AG) and anandamide (AEA) are believed to be the most important) and phytocannabinoids such as cannabidiol (CBD) or tetrahydrocannabinol (THC) [13,14]. Cannabinoids are believed to be negative regulators of inflammation, so an increased activation of cannabinoid receptors is very often viewed as a cellular protective mechanism [15]. Nevertheless, the impact of phytocannabinoids on apoptosis is not entirely clear. In some cells, cannabinoids are pro-apoptotic, while in others they show anti-apoptotic effects [16,17]. There are two main hypotheses explaining this discrepancy. The first one is based on the differences in the expression of endocannabinoid receptors between cells, as agonists and antagonists of these receptors have opposite effects on apoptosis [18,19]. Nevertheless, recent studies have shown that cells with higher COX-2 activity are more prone to apoptosis following the administration of different cannabinoids. COX-2, independently of AA metabolism, can also metabolize cannabidiol and anandamide to prostaglandin derivatives, which are believed to have pro-apoptotic properties [20].

Moreover, COX-2 activity is usually higher in inflammation caused by disease (i.e., autoimmune diseases, cancer) or by exogenous factors such as UVB radiation. Therefore, it is believed that other factors, such as an increased expression of cytokines or an increased production of ROS may also influence apoptosis [21]. For this reason, it is difficult to clearly determine whether interactions between COX-2 and cannabinoids are responsible for the increased apoptosis of these cells, or whether this is mainly caused by other factors [22,23]. This seems to be a particularly important issue as cannabinoids are considered to be promising agents in the treatment of various diseases and conditions, including various skin conditions—the skin is the outer layer of the body, being particularly sensitive to exogenous factors. Therefore, the aim of this study is to evaluate the COX-2-inhibitory pathway of celecoxib and to relate it to the apoptosis of UV-irradiated keratinocytes treated with AEA or CBD.

## 2. Materials and Methods

Primary Epidermal Keratinocytes; (Normal, Human, Adult; ATCC^®^ Number: PCS-200-011) were obtained from American Type Culture Collection, (ATCC, Manassa, VA, USA; https://www.atcc.org/products/pcs-200-011; accessed 10 May 2022). Cells were grown under standard conditions in Dulbecco’s Modified Eagle’s Medium (DMEM) with 10% fetal bovine serum (FBS) supplemented with 50 U/mL penicillin and 50 μg/mL streptomycin (humidified atmosphere of 5% CO_2_ at 37 °C) until the 4th passage. When cells reached 90% of confluency they were used for experiment. The inducible form of COX (COX-2) could modify the keratinocyte apoptosis process. The evaluation of the effect of this enzyme was carried out using the COX-2 inhibitor celecoxib at a final concentration of 20 µM, which is known to be enough to inhibit COX-2 action in living cells [24]. Another assumption concerned the role of cannabinoids in the regulation of the apoptosis process. In order to evaluate the effects of the phytocannabinoid cannabidiol (CBD) and the endocannabinoid–anandamide (AEA), these compounds were used with a final concentration of 4 µM, the concentration that is enough to cause biochemical changes as well as apoptosis of cells [25]. For that purpose, culture media were replaced and supplemented with the abovementioned compounds or with non-supplemented in the case of control cells.

**Figure 1:**
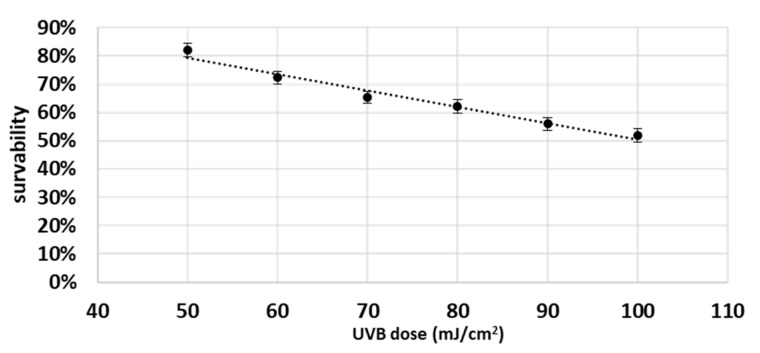
Survival curve of keratinocytes after UVB irradiation. The results show the survival percentage of cells irradiated with UVB compared to non-irradiated cells.

Moreover, as UVB activates both COX-2 and apoptosis, it is very likely that COX-2 plays an important role in the regulation of apoptosis by UVB. Therefore, cells were UVB irradiated at a distance 15 cm from 6 lamps, 6W each, and total UV dose was 60 mJ/cm^2^ (Bio-Link Crosslinker BLX, Vilber Lourmat, Eberhandzel, Germany). The radiation was followed by replacement of medium with cold PBS (4 °C), and after radiation PBS was replaced with medium and cells were incubated for 24 h. In order to select the dose of UVB radiation, preliminary studies were carried out. For this purpose, cells were grown in 24-well plates for 24 h and then exposed to different doses of UVB radiation (from 50 to 100 mJ/cm^2^) (on ice). The cells were then washed with PBS and then incubated for 10 min at 37 °C with 500 µL of (0.5%) MTT solution. After removing MTT solution, 800 µL of DMSO was added to the wells, and after 3 min the absorbance was read at 570 nm. Figure 1 shows the survival curve of keratinocytes. A dose was selected that reduced the survival of keratinocytes to about 70% (0.06 J/cm^2^).

When cells were treated with both UVB and chemical compound(s), they were pretreated with this compound(s) for 2 hours, irradiated with UVB as detailed mentioned, and then PBS was replaced with medium supplemented with chemical factors. Every group was incubated for 24 h.

Experimental groups were divided into 2 main groups:

A—keratinocytes not treated with celecoxib

B—keratinocytes treated with 20 µM celecoxib to inhibit COX-2 activity.

These two main groups were divided into the following subgroups:

- **Control**—cells cultured for 24 in standard medium without (A) or with 20 µM celecoxib (B);
- **CBD**—cells were pre-cultured with 4 µM CBD and then cultured for 24 in standard medium without (A) or with 20 µM celecoxib (B);
- **AEA**—cells were pre-cultured with 4 µM AEA and then cultured for 24 in standard medium without (A) or with 20 µM celecoxib (B);
- **UVB**—cells were irradiated with UVB (60 mJ/cm^2^), and then cultured for 24 in standard medium without (A) or with 20 µM celecoxib (B);
- **CBD + UVB**—cells were pre-cultured for 24 h with 4 µM CBD, next irradiated with UVB (60 mJ/cm^2^), and then cultured for 24 h in medium supplemented with 4 µM CBD. The last 24 h culturing was carried out in medium without (A) or with 20 µM celecoxib (B);
- **AEA + UVB**—cells were pre-cultured for 24 h with 4 µM with AEA, irradiated with UVB (60 mJ/cm^2^), and then cultured for 24 h in medium supplemented with 4 µM AEA. The last 24 h culturing was carried out in medium without (A) or with 20 µM celecoxib (B).

After incubation, some of the cells were frozen at −80 °C for examination of COX-2 activity. The rest of the cells were used immediately for RNA extraction and microscopic examination. Post-culture media were also frozen and used for prostaglandins determination. Every examination was performed on 3 independent cell cultures.

### 2.1. Assessment of Apoptosis Regulators

Transcription of caspases 2,8,9; Bcl2; Bax; CB1; CB2; TNFα were analyzed using a 2-step quantitate reverse-transcriptase polymerase chain reaction (qRT-PCR) method preceded by mRNA isolation from fresh cells using the column-isolation method (EXTRACTME RNA isolation kit; BLIRT S.A. Gdańsk, Poland) according to manufacturer’s instruction. This method is based on spin mini columns with membranes, which efficiently and selectively bind nucleic acids at high concentrations of chaotropic salts. During the first isolation step, RNA from cells were bound to a Purification Column membrane by the addition of ethanol. A 3-time washing stage effectively removed impurities and enzyme inhibitors. Purified RNA was eluted with the use of RNase-free water. RNA levels were measured using theNano-Drop system (NanoDrop™ 2000/2000c; Thermo Fisher Scientific, Inc., Cleveland, OH, USA) [26].

After that, 40 μg of RNA were used and reverse transcription reaction was performed to obtain cDNA (iScript™ Advanced cDNA Synthesis Kit, Bio-Rad Laboratories Inc., Hercules, CA, USA) with the following protocol: priming 5 min, 25 °C, reverse transcription 20 min 46 °C, reverse transcriptase inactivation 1 min 95 °C on Agilent Mx3000P qPCR thermocycler (Agilent Santa Clara, CA, USA).

In the next step, quantitative polymerase chain reaction was performed. Specific primers for caspases 2,8,9; Bcl2; Bax; CB1; CB2; TNFα were designed by BIO-RAD (Bio-Rad Laboratories Inc., Hercules, CA, USA). The housekeeping gene GAPDH was used as the reference gene for quantification. Reaction protocol was: denaturation and polymerase activation: 30 s; 95 °C, then in 40 cycles: denaturation 5 s 95 °C; annealing, extension and read 30 s 60 °C in total reaction volume of 20 μL (SsoAdvanced Universal SYBR Green Supermix; Bio-Rad Laboratories Inc., Hercules, CA, USA) on Agilent Mx3000P qPCR thermocycler (Agilent Santa Clara, CA, USA). Melting curve analysis was performed to check if non-specific products had not been formed. The relative quantification of gene expression was determined by comparison of values of Ct using the ΔΔCt method. All results were normalized to GAPDH expression [27].

### 2.2. Assessment of the Number of Cells Undergoing Apoptosis

Percentage of apoptotic cells was examined immediately after culture in fresh cells using Annexin V-FITC Apoptosis Kit (BioVision, Inc., Milpitas, CA, USA) according to manufacturer instructions on a Nikon Eclipse Ti (Nikon, Tokyo, Japan) fluorescent microscope equipped with standard filters. Then, 500,000 cells were resuspended in binding buffer and 5 μL of Annexin V-FITC and propidium iodide were added. Cells were incubated for 5 min in the dark (room temperature), and then were transferred to a glass slide and covered with a glass coverslip. Cells were observed under 40× magnification using corresponding filters. Annexin V binds to phosphatidylserine, which undergoes externalization during apoptosis; cells stained with annexin were recognized as an apoptotic, otherwise as not. The total amount of cells counted was 200, and the percentage of apoptotic cells was then calculated [28].

### 2.3. Measurement of Cyclooxygenase Activity

Cyclooxygenase 2 (COX1/2-EC.1.14.99.1) activity was measured spectrophotometrically using a commercial assay kit (Cayman Chemical Company, Ann Arbor, MI, USA) [29]. Prior to examination cells were sonicated and centrifugated on 10,000× *g* at 4 °C for 15 min. The supernatant was determined by the colorimetric assay based on measurement of formation of oxidized N,N,N’,N’-Tetramethyl-p-phenylenediamine dihydrochloride from colorimetric substate (included in the kit) at 590 nm. The specific COX1-inhibitor SC-560 (included in the kit) was applied to measure only COX-2 activity [30].

### 2.4. Determination of Prostaglandins Level in Culture Media

Prostaglandin E2 (PGE2), Prostaglandin D2 (PGD2) and Prostaglandin E2 Ethanolamide (PGE2-EA) were determined using liquid chromatography tandem mass spectrometry (LC-MS/MS) (LC-MS 8060, Shimadzu, Kyoto, Japan). The culture medium was used to determine the level of prostaglandins (PGD2, PGE2 and PGE2-EA). First, 20 µL of d4-PGD2 and d4-PGE2-EA (100 ng/mL each) were added 500 µL of medium samples as internal standards. Then PGE2, PGD2 and PGE2-EA were extracted with 2 mL of hexane/ethyl acetate mixture (1:1, *v*/*v*). After centrifugation at 4 °C, 10,000× *g* for 15 min, the upper organic layer was collected and saved for examination. For each sample, extraction was performed 3 times and all organic phases for a given sample were pooled. The organic phases were evaporated to dryness under a nitrogen stream and dissolved in 200 μL of acetonitrile [31]. Prostaglandins were separated on a ZorBax Eclipse Plus C18 analytical column (2.1 × 100 mm, 1.8 µm particle size). Electrospray ionization (ESI) in negative (PGE2 and PGD2) and positive (PGE2-EA) modes was used for multiple reaction monitoring (MRM) and quantification of analytes. The precursors to the product ion transition was as follows: *m*/*z* 351.3→271.20 for PGE2 and PGD2, *m*/*z* 396.3→378.15 for PGE2-EA, *m*/*z* 355.0→275.25 for PGD2-d4 and *m*/*z* 400.3→382.3 for PGE2-EA-d4 [32]. Results were calculated and presented pmol per mL of culture medium.

### 2.5. Statistical Analysis

Statistical analysis between groups was performed using a one-way ANOVA test (using Statistica 13.3 software), and statistically significant differences for *p* < 0.05 are shown in the figures.

## 3. Results

**Figure 2:**
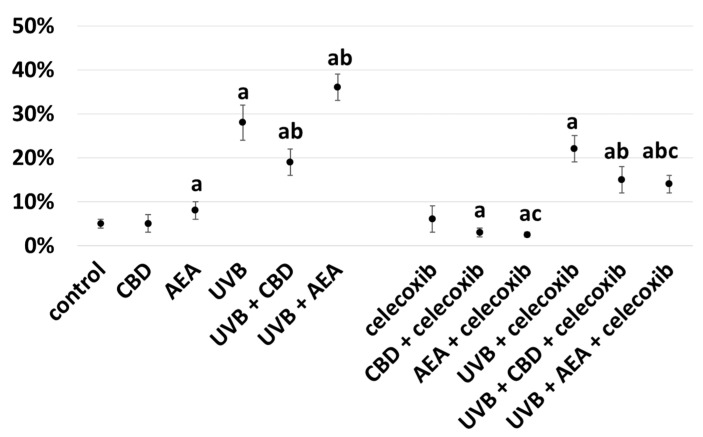
Percentage of cells undergoing apoptosis in control keratinocytes treated with CBD (4 μM) or AEA (4 μM) or irradiated with UVB (60 mJ/cm^2^) as well as cells irradiated with UVB and treated with CBD or AEA (in the same concentrations), as well as their expression in keratinocytes after treatment with 20 μM celecoxib alone and with CBD(4 μM) or AEA(4 μM) as well as cells irradiated with UVB and treated with CBD or AEA (in the same concentrations). (a)—statistically significant differences between keratinocytes treated with CBD, AEA, UVB, UVB + CBD, AEA + UVB and control cells as well as keratinocytes treated with CBD + celecoxib, AEA + celecoxib, UVB + celecoxib, UVB + CBD + celecoxib and AEA + UVB+ celecoxib and cells treated only with celecoxib; *p* < 0.05; (b)—statistically significant differences between keratinocytes treated with UVB + CBD, UVB + AEA, UVB + CBD + celecoxib, AEA + UVB + celecoxib and irradiated with UVB *p* < 0.05; (c)—statistically significant differences between keratinocytes treated with celecoxib and corresponding keratinocytes not treated with celecoxib.

CBD inhibited apoptosis of keratinocytes, which was observed in celecoxib-treated and untreated cells and simultaneously irradiated with UVB (Figure 2). Moreover, inhibition of COX-2 by celecoxib increased anti-apoptotic properties of cannabidiol, as in every examined case in keratinocytes treated with celecoxib, apoptosis was lower than in not-treated cells. In contrary, anandamide increased apoptosis in cells not treated with celecoxib but decreased it in those treated, suggesting that COX-2 activity had a higher impact on anandamide action than on CBD action. Additionally, inhibition of COX-2 partially ameliorated pro-apoptotic properties of UVB.

**Figure 3:**
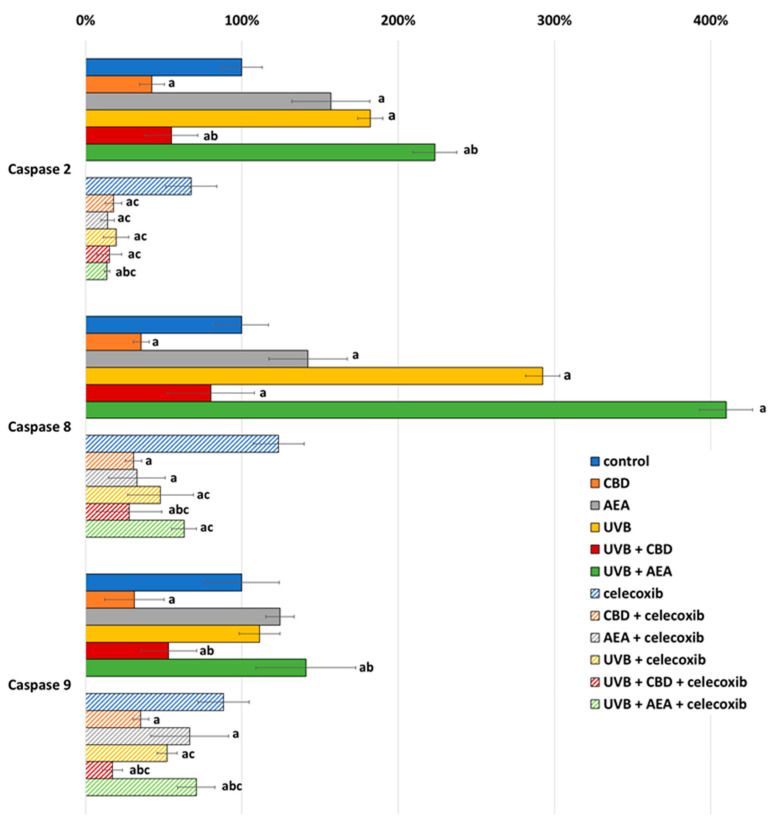
Caspase expression in control keratinocytes treated with CBD (4 μM) or AEA (4 μM) or irradiated with UVB (60 mJ/cm^2^) as well as cells irradiated with UVB and treated with CBD or AEA (in the same concentrations), as well as its expression in keratinocytes after treatment with 20 μM celecoxib alone and with CBD(4 μM) or AEA(4 μM) as well as cells irradiated with UVB and treated with CBD or AEA (in the same concentrations). (a)—statistically significant differences between keratinocytes treated with CBD, AEA, UVB, UVB + CBD, AEA + UVB and non-treated cells as well as keratinocytes treated with CBD + celecoxib, AEA + celecoxib, UVB + celecoxib, UVB + CBD + celecoxib and AEA + UVB+ celecoxib and cells treated only with celecoxib; *p* < 0.05; (b)—statistically significant differences between keratinocytes treated with UVB + CBD, UVB + AEA, UVB + CBD + celecoxib, AEA + UVB + celecoxib and irradiated with UVB *p* < 0.05; (c)—statistically significant differences between keratinocytes treated with celecoxib and corresponding keratinocytes not treated with celecoxib.

The anti-apoptotic properties of cannabidiol may result from reduction of the expression of caspases 2, 8 and 9 (which are initiative caspases for all three main apoptotic pathways), which was observed in both UVB-irradiated and non-irradiated keratinocytes regardless of celecoxib treatment (Figure 3). Moreover, the expression of all of these caspases was lower in UVB-irradiated cells treated with celecoxib compared to those that were only irradiated with UVB. Moreover, in cells not treated with celecoxib, caspase expression was increased by AEA, but in celecoxib-treated keratinocytes, AEA decreased the expression of these enzymes. In the case of UVB-irradiated cells, only caspase 2 expression was reduced by AEA treatment.

**Figure 4:**
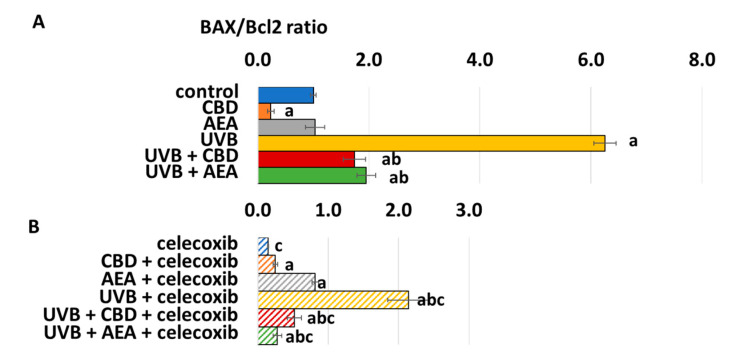
(**A**) BAX/Bcl2 ratio in control keratinocytes treated with CBD (4 μM) or AEA (4 μM) or irradiated with UVB (60 mJ/cm^2^) as well as cells irradiated with UVB and treated with CBD or AEA (in the same concentrations). (**B**) BAX/Bcl2 ratio in keratinocytes after treatment with 20 μM celecoxib alone and with CBD(4 μM) or AEA(4 μM) or irradiated with UVB (60 mJ/cm^2^) as well as cells irradiated with UVB and treated with CBD or AEA (in the same concentrations). (a)—statistically significant differences between keratinocytes treated with CBD, AEA, UVB, UVB + CBD, AEA + UVB and non-treated cells as well as keratinocytes treated with CBD + celecoxib, AEA + celecoxib, UVB + celecoxib, UVB + CBD + celecoxib and AEA + UVB+ celecoxib and cells treated only with celecoxib; *p* < 0.05; (b)—statistically significant differences between keratinocytes treated with UVB + CBD, UVB + AEA, UVB + CBD + celecoxib, AEA + UVB + celecoxib and irradiated with UVB *p* < 0.05; (c)—statistically significant differences between keratinocytes treated with celecoxib and corresponding keratinocytes not treated with celecoxib.

Since Bcl2 is one of the strongest inhibitors of apoptosis while BAX is one of the most important apoptosis activators, their ratio significantly influences the pro- or anti-apoptotic tendency in cells. This was especially important in the case of UVB-treated cells where both CBD and AEA decreased the Bax/Bcl ratio, leading to lower caspase transcription and decreased apoptosis. On the contrary, UVB dramatically increased the Bax/Bcl2 ratio, as shown on Figure 4.

**Figure 5:**
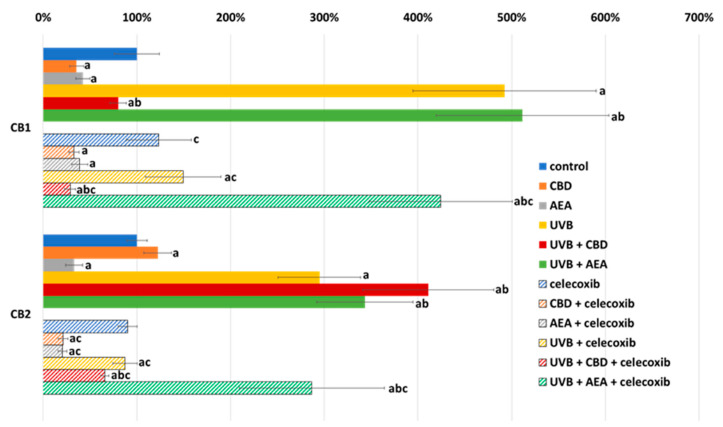
Cannabinoid receptors (CB1 and CB2) expression in control keratinocytes treated with CBD (4 μM) or AEA (4 μM) or irradiated with UVB (60 mJ/cm^2^) as well as cells irradiated with UVB and treated with CBD or AEA (in the same concentrations) as well as their expression in keratinocytes after treatment with 20 μM celecoxib alone and with CBD(4 μM) or AEA(4 μM) as well as cells irradiated with UVB and treated with CBD or AEA (in the same concentrations). (a)—statistically significant differences between keratinocytes treated with CBD, AEA, UVB, UVB + CBD, AEA + UVB and non-treated cells as well as keratinocytes treated with CBD + celecoxib, AEA + celecoxib, UVB + celecoxib, UVB + CBD + celecoxib and AEA + UVB+ celecoxib and cells treated only with celecoxib; *p* < 0.05; (b)—statistically significant differences between keratinocytes treated with UVB + CBD, UVB + AEA, UVB + CBD + celecoxib, AEA + UVB + celecoxib and irradiated with UVB *p* < 0.05; (c)—statistically significant differences between keratinocytes treated with celecoxib and corresponding keratinocytes not treated with celecoxib.

The action of cannabinoids is dependent on their interaction with receptors, the expression of which was increased by UVB. Anti-apoptotic and anti-inflammatory CB2 was about three-fold and pro-apoptotic and pro-inflammatory CB1 about five-fold higher than in the control. This suggests that pro-inflammatory signaling dominates in this case, which may partly explain the pro-inflammatory properties of UVB (Figure 5). Additionally, AEA tended to reduce the activation of cannabinoid receptors in untreated keratinocytes, while in UVB irradiated cells AEA acted the opposite way. On the other hand, cannabidiol increased anti-inflammatory CB2 expression, but decreased pro-inflammatory CB1.

**Figure 6:**
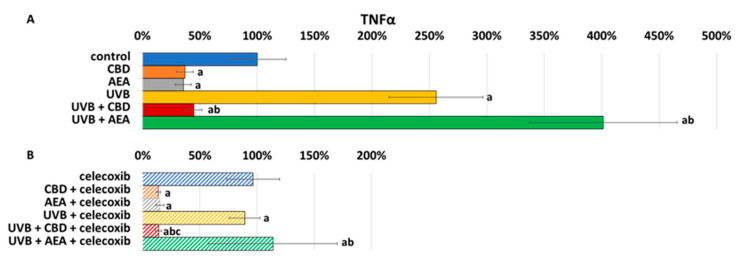
(**A**) TNFα expression in control keratinocytes treated with CBD (4 μM) or AEA (4 μM) or irradiated with UVB (60 mJ/cm^2^) as well as cells irradiated with UVB and treated with CBD or AEA (in the same concentrations). (**B**) TNFα expression in keratinocytes after treatment with 20μM celecoxib alone and with CBD(4 μM) or AEA(4 μM) or irradiated with UVB (60 mJ/cm^2^) as well as cells irradiated with UVB and treated with CBD or AEA (in the same concentrations). a—statistically significant differences between keratinocytes treated with CBD, AEA, UVB, UVB + CBD, AEA + UVB and non-treated cells as well as keratinocytes treated with CBD + celecoxib, AEA + celecoxib, UVB + celecoxib, UVB + CBD + celecoxib and AEA + UVB + celecoxib and cells treated only with celecoxib; *p* < 0.05; b—statistically significant differences between keratinocytes treated with UVB + CBD, UVB + AEA, UVB + CBD + celecoxib, AEA + UVB + celecoxib and irradiated with UVB *p* < 0.05; c—statistically significant differences between keratinocytes treated with celecoxib and corresponding keratinocytes not treated with celecoxib.

As CB1/2 receptors are important regulators of inflammation, changes in their expression may result in different TNFα synthesis Figure 6. In cells treated with celecoxib, a lower expression of TNFα indicates that inhibition of COX-2 ameliorated oxidative stress and inflammation. On the other hand, UVB increased TNFα expression. However, CBD reveals anti-inflammatory and antioxidant properties, while the effects of AEA appear to be highly dependent on UVB radiation, as in this case only AEA shows positive impact.

**Figure 7:**
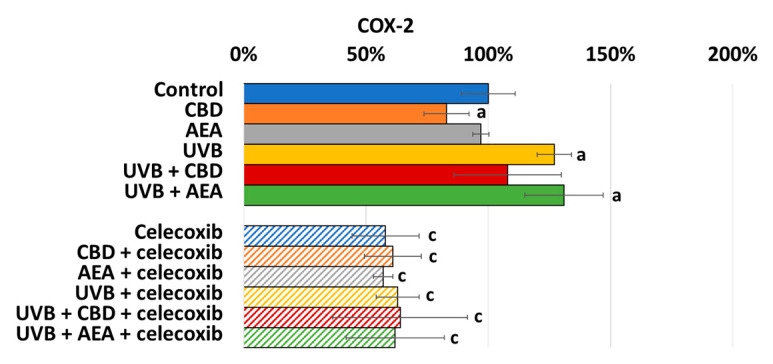
COX-2 activity in control keratinocytes treated with CBD (4 μM) or AEA (4 μM) or irradiated with UVB (60 mJ/cm^2^) as well as cells irradiated with UVB and treated with CBD or AEA (in the same concentrations) as well as its expression in keratinocytes after treatment with 20 μM celecoxib alone and with CBD(4 μM) or AEA(4 μM) as well as cells irradiated with UVB and treated with CBD or AEA (in the same concentrations). (a)—statistically significant differences between keratinocytes treated with CBD, AEA, UVB, UVB + CBD, AEA + UVB and non-treated cells as well as keratinocytes treated with CBD + celecoxib, AEA + celecoxib, UVB + celecoxib, UVB + CBD + celecoxib and AEA + UVB+ celecoxib and cells treated only with celecoxib; *p* < 0.05; (b)—statistically significant differences between keratinocytes treated with UVB + CBD, UVB + AEA, UVB + CBD + celecoxib, AEA + UVB + celecoxib and irradiated with UVB *p* < 0.05; (c)—statistically significant differences between keratinocytes treated with celecoxib and corresponding keratinocytes not treated with celecoxib.

Intensification of inflammation leads to activation of COX-2. As shown in Figure 7, COX-2 activity is higher in the UVB-radiated group, and its activity is slightly decreased by CBD but not AEA. Of course, the celecoxib addition leads to a decrease in COX-2 activity.

**Figure 8:**
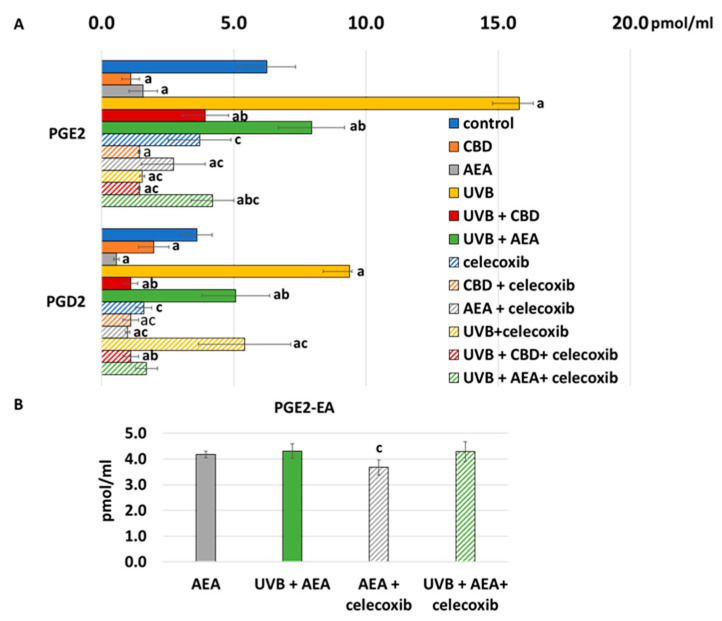
(**A**) Prostaglandins level in culture medium of control keratinocytes treated with CBD (4 μM) or AEA (4μM) or irradiated with UVB (60 mJ/cm^2^) as well as cells irradiated with UVB and treated with CBD or AEA (in the same concentrations) as well as their levels in keratinocytes after treatment with 20 μM celecoxib alone and with CBD(4 μM) or AEA(4 μM) as well as cells irradiated with UVB and treated with CBD or AEA (in the same concentrations). (a)—statistically significant differences between keratinocytes treated with CBD, AEA, UVB, UVB + CBD, AEA + UVB and non-treated cells as well as keratinocytes treated with CBD + celecoxib, AEA + celecoxib, UVB + celecoxib, UVB + CBD + celecoxib and AEA + UVB+ celecoxib and cells treated only with celecoxib; *p* < 0.05; (b)—statistically significant differences between keratinocytes treated with UVB + CBD, UVB + AEA, UVB + CBD + celecoxib, AEA + UVB + celecoxib and irradiated with UVB *p* < 0.05; (c)—statistically significant differences between keratinocytes treated with celecoxib and corresponding keratinocytes not treated with celecoxib. (**B**) PGE2-EA levels in culture medium of control keratinocytes and treated with AEA (4 μM) or irradiated with UVB (60 mJ/cm^2^) and treated with AEA (4 μM) or treated with 20 μM celecoxib and AEA (4 μM) or irradiated with UVB and treated with AEA and celecoxib in above abovementioned doses.

As COX-2 is one of the main enzymes responsible for the synthesis action of prostaglandin, an increase in celecoxib (which is its inhibitor) led to much lower generation of prostaglandins than without it, as shown at Figure 8. Also, CBD led to lower COX-2 activity and lower generation of prostaglandins. On the other hand, AEA did not influence COX-2 activity, but as it is also a substrate for COX-2, it competed with AA. Therefore, in this case, generation of PGE2 and PGD2 was lower, but PGE2-EA was also present. UVB, as a known pro-inflammatory factor, increased both COX-2 activity and prostaglandin generation.

## 4. Discussion

In any multicellular organism, apoptosis plays an important role in maintaining homeostasis. Nevertheless, the process of apoptosis may be disrupted by various factors, such as UVB radiation, inflammation, or changes in levels of endo- as well as exogenous cannabinoids, including phytocannabinoids. The action of these factors activates various complex metabolic pathways. In addition, the same factor may act in a pro-apoptotic way in one case, while in another the action may be anti-apoptotic. This situation applies, inter alia, to cannabinoids that induce apoptosis in some cases and act cytoprotectively in others [33,34,35]. It is believed that this may be due to the different expression of the receptors as well as the different activities of enzymes and regulatory proteins in different cells. On the other hand, UVB radiation is considered as a pro-apoptotic factor, but its exact mechanism of action is not fully understood [36,37].

Endocannabinoids are physiologically produced in the body but various factors, such as UVB, may disrupt their generation. On the other hand, exocannabinoids are believed to be potential medicinal agents used in the therapy of diseases in which apoptosis is dysregulated. Still, both endo- and exocannabinoids may act in a pro- or an anti-apoptotic manner, depending on the targeted cells [33,34,35]. It is believed that this may be due to the different expression of receptors as well as the different activities of enzymes such as COX-2 and the regulatory proteins in different cells. On the other hand, UVB activates COX-2, as shown in this study, so it is possible that this enzyme is the link between the impact of UVB radiation and that of cannabinoids on apoptosis. Still, COX-2 is also important in the regulation of inflammation, as it produces prostaglandins, which are known to be important pro-inflammatory factors.

### 4.1. Effect of UVB on Keratinocyte Apoptosis

Apoptosis can be activated on three main pathways, with UVB radiation activating all of them [38,39]. To activate the intrinsic apoptotic pathway, it is necessary for BAX to interact with the mitochondrial membrane, but this interaction is blocked by the anti-apoptotic protein Bcl2 [11]. Therefore, the Bax/Bcl2 ratio is critical in the regulation of the intrinsic pathway. As UVB radiation changes this ratio in favor of Bax, it promotes the activation of the intrinsic pathway of apoptosis, thus an increased transcription of caspase 9, believed to be the marker of this pathway, has been observed in this study. On the other hand, caspase 8 can mainly be activated by the so-called death ligands, which, by interacting with their receptors, activate the extrinsic pathway. As one of the main death ligands is TNFα, its observed increased expression not only indicates inflammation but also leads to the activation of the extrinsic pathway of apoptosis. The ER stress pathway is usually activated by ROS-dependent disturbances in protein metabolism; however, ER stress does not always lead to apoptosis as many cytoprotective mechanisms are also activated during its course to alleviate stress. These protective actions include increased transcription of Nrf2, which stimulates the transcription of ARE-dependent cytoprotective genes such as MDM2 or Bcl-2 [40,41]. Yet, as shown in this study, Bcl2 expression is not increased, suggesting an impairment of the protective mechanism, which in consequence leads to the observed increased expression of caspase 2.

UVB radiation also influences cannabinoid signaling, as its action leads to an increased expression of both CB1 and CB2 receptors; nevertheless, an increase in CB1 expression is more potent than in the case of CB2. As CB1 is believed to be a pro-inflammatory and pro-apoptotic receptor [42], an increase in its expression further enhances apoptosis and inflammation.

All the changes mentioned above seem to be at least partially modulated by COX-2. The action of COX-2 leads to the production of various lipid metabolites such as PGE2 and PGD2. PGE2 is known to stimulate Bcl-2 expression, while PGD2 acts in the opposite manner [43]. On the other hand, UVB radiation leads to an increased production of both of these prostaglandins. However, the shift in the Bax/Bcl2 balance in favor of Bax suggests that the pro-apoptotic properties of PGD2 dominate, being one of the reasons for the increased keratinocyte apoptosis following UVB irradiation. Inhibition of COX-2 activity by celecoxib inhibits the generation of the aforementioned prostaglandins, which partially restores the Bax/Bcl2 balance, thus reducing apoptosis. Still, COX-2 also plays an important role in regulating inflammation by modeling prostaglandin levels. Nevertheless, celecoxib can induce the mitochondrial apoptotic pathway independently of its action on cyclooxygenase [44]. As in non-irradiated cells COX-2 activity is low, its further inhibition has no effect on apoptosis, so pro-apoptotic properties of celecoxib lead to increased apoptosis. This effect is not observed in irradiated cells, in which the impact of celecoxib on COX-2 seems to be more important than its impact on the mitochondrial apoptotic pathway.

Prostaglandins act in a paracrine manner and may induce pro-inflammatory changes not only directly in the cell in which they were produced, but also in others. As their increased levels have been observed in post-culture media, it is highly likely that they are secreted from keratinocytes in the human body as well and interact with skin-infiltrating leukocytes. Prostaglandins are especially important as regulators of the differentiation of lymphocytes into subpopulations. Mice with knockout prostaglandin E receptor 1 (EP1) show a diminished Th1 response, suggesting that PGE2 is also necessary for the development of a Th1 response [45]. Moreover, EP1 agonists in vitro promote Th1 responses [45], while PGE2 in vivo induces Th17 cell differentiation, as well as the production of pro-inflammatory cytokines [46]. On the other hand, inhibition of COX-2 by non-steroidal anti-inflammatory drugs inhibits the development of Th1 and Th2 lymphocytes [47]. Moreover, in vitro COX-2 inhibition reduces B cell maturation and antibody production, with EP2 and EP4 receptors playing the most important role in the process as their agonists affect B cell activation [48,49]. Similarly, as shown in another study, the inhibition of COX-2 leads to a reduced secretion of prostaglandins by keratinocytes, as well as a decreased transcription of TNFα, thus reducing inflammation.

### 4.2. Effect of Cannabinoids on Apoptosis

It is known that cannabinoids (both phytocannabinoids and endocannabinoids), including CBD and AEA, can act either as pro- or anti-apoptotic factors, depending on the type of cells, their receptors, and the metabolic profile [21]. In some cells, such as cardiomyocytes and neurons, CBD and AEA protect against apoptosis [50,51,52]. However, in both immune and neoplastic cells, they induce apoptosis [34,53,54]. As was observed for chorionic cells (BeWo), AEA intensifies apoptosis by activating both initiator caspase 9 and executive caspases 3 and 7 [55]. The cardioprotective drug propofol was found to cause a significant increase in the release of endocannabinoids (AEA and 2-AG) by cardiomyocytes. This was accompanied by an increased activation of CB1 and CB2 receptors at both mRNA and protein levels, while the cytoprotective effect of propofol is abolished by CB2 antagonists, although not by CB1 antagonists [52].

In the present study, CBD and AEA seem to act in opposite manners on cells with normal COX-2 activity, yet their effect is similar in the case of cells with celecoxib-induced COX-2 inhibition. CBD does not change the percentage of cells undergoing apoptosis, even though it decreases the expression of caspases 3, 8, and 9 in the case of keratinocytes otherwise untreated. These results confirm the previously observed protective properties of cannabidiol [56]. However, a stronger inhibition of caspase expression is observed in the case of celecoxib-treated cells in which cannabidiol also decreases the number of cells undergoing apoptosis. It can therefore be stated that the effect of cannabidiol on apoptosis is modulated by COX-2, or by CB1 and CB2 signaling. Nevertheless, cannabidiol may induce apoptosis both in the case of cells with a dominant CB1 receptor and in those with a dominant CB2 receptor [7], and antagonists of these receptors abolish the pro-apoptotic effect of CBD [18]. Nevertheless CBD, being only a partial CB2 receptor agonist may not cause as potent an anti-apoptotic effect as its full agonists [57]. It is believed that activation of any of these receptors may lead to apoptosis. On the other hand, the latest studies suggest that CB2 agonists shows cytoprotective effects, which means that in some cases CB2 signaling is involved in this process [58]. In the present study, a reduced expression of cannabinoid receptors in the case of CB1 and CB2 signaling is observed for celecoxib-treated cells; however, in the case of untreated cells, only CB1 expression is reduced after CBD administration. This seems to correlate with the decreased apoptosis after CBD administration found only in celecoxib-treated keratinocytes. Another explanation is the different activity of COX-2. In cancer cells, in which cannabidiol normally acts as a pro-apoptotic factor, COX-2 inhibition leads to the inhibition of apoptosis, which is probably caused by the lower generation of PGD2 [59]. In this study, CBD causes a slight reduction in COX-2 activity, leading to a lower generation of prostaglandins, especially PGD2, which reduces the Bax/Bcl2 ratio, finally causing a slight decrease in the activation of caspases. Nevertheless, a combination of celecoxib and CBD leads to a further decrease in the level of pro-apoptotic PGD2, which results in a more potent inhibition of apoptosis. Additionally, celecoxib and CBD act as anti-inflammatory factors, due to the reduced expression of TNFα.

Unlike CBD, the use of anandamide enhances the expression of caspases 2 and 8 and increases the number of apoptotic cells among non-UVB-irradiated cells. The increased keratinocyte apoptosis following AEA treatment was also observed in previous studies [60]. As caspase 9 remains unchanged in non-radiated cells and is only slightly increased in the case of AEA administration after UVB irradiation, this suggests that the intrinsic pathway is not affected by anandamide, only the extrinsic and ER-stress pathways are induced. It is known that through the activation of CB1 receptors, AEA increases ROS generation, which leads to the activation of MAPK p38 and enhances the generation of TNFα, which, as one of the death ligands, activates the extrinsic pathway [61,62]. Nevertheless, in the present study, TNFα expression did not correspond with caspase 8 expression. This suggests that AEA does not act as a pro-inflammatory factor, only as a pro-apoptotic one. However, the expression of other death ligands, such as TRAIL or CD95L, may also be enhanced by MAPK, which leads to the activation of the extrinsic pathway [63]. In addition, AEA may induce autophagy and ER stress. The activation of cannabinoid receptors by AEA leads to an increased generation of ceramides, previously identified in the form of an increased PERK phosphorylation, increased IRE1 activity, and ATF6 translocation to the cell nucleus after the administration of endocannabinoids [64,65,66]. In this study, the activation of the ER stress-induced pathway by AEA is confirmed by the increased caspase 2 expression. Nevertheless, all the aforementioned changes are observed only in cells untreated with celecoxib. In celecoxib-treated cells, AEA, similarly to cannabidiol, acts as a cytoprotective factor, its action leading to decreased apoptosis and reduced caspases expression. This is probably caused by AEA competing with AA, as they are both COX-2 substrates. Therefore, COX-2 metabolizes AEA rather than arachidonic acid, which in consequence leads to PGE2-EA production [67]. As PGE2-EA is also a pro-apoptotic compound, its generation leads to increased apoptosis, possibly also by altering the Bax/Bcl2 ratio in cells without celecoxib. Nevertheless, in the case of cells treated with celecoxib, the generation of PGE2-EA, as well as that of other pro-apoptotic factors, is lower; hence, apoptosis is decreased. As COX-2 action is pro-inflammatory, its inhibition decreases TNFα expression and since the cytokine is one of the most important death ligands, its decreased generation leads to a lower activation of the extrinsic pathway.

CBD is also cytoprotective in UVB-irradiated cells. On the other hand, the changes induced by AEA in keratinocytes are further exacerbated, triggering the activation of the intrinsic apoptotic pathway by AEA. This pathway is mainly regulated by the Bax/Bcl2 ratio, while in the case of non-irradiated cells, AEA does not affect their balance. However, in the case of irradiated cells, AEA shifts the balance in favor of Bax, which may be the cause of the intrinsic pathway activation. Moreover, in this case, celecoxib administration ameliorates the pro-apoptotic properties of AEA, as the lower generation of prostaglandins leads to the restoration of the Bax/Bcl2 balance and a decreased apoptosis. As TNFα expression is also decreased, this suggests that both the intrinsic and the extrinsic pathways are reduced, as is inflammation. There is also a significant difference between the actions of CBD and AEA in the case of their influence on cannabinoid receptors. In non-irradiated cells, both cannabinoids reduce CB1 expression, but AEA also reduces CB2 signaling, while in irradiated cells, AEA increases CB1 and CB2 expression, especially following the administration of celecoxib.

## 5. Conclusions

The pro-apoptotic and pro-inflammatory effect of UVB is partly dependent on COX-2 activity and prostaglandin production since inhibition of COX-2 activity by celecoxib not only reduces prostaglandin generation but also decreases the expression of apoptotic activators and TNFα level compared to UVB-irradiated keratinocytes. As a consequence, apoptosis of these cells is also reduced. The same is the case with AEA, which also induces keratinocyte apoptosis by increasing prostaglandin synthesis as well as activating caspases.

On the other hand, COX inhibition by celecoxib leads to a reduced prostaglandin synthesis in AEA-treated cells and a decreased generation of PGE2-EA, an AEA metabolite. Reduced apoptosis and inflammation are also observed in these cells, suggesting that the action of COX-2 is necessary for the pro-apoptotic and pro-inflammatory action of AEA. Still, the anti-apoptotic and anti-inflammatory action of CBD seems to be independent from COX activity.

## Data Availability

Not applicable.
